# Localization of Mobile Robots Using Odometry and an External Vision Sensor

**DOI:** 10.3390/s100403655

**Published:** 2010-04-13

**Authors:** Daniel Pizarro, Manuel Mazo, Enrique Santiso, Marta Marron, David Jimenez, Santiago Cobreces, Cristina Losada

**Affiliations:** Department of Electronics, University of Alcala, NII km 33,600, Alcala de Henares, Spain; E-Mails: mazo@depeca.uah.es (M.M.); santiso@depeca.uah.es (E.S.); marta@depeca.uah.es (M.M.); david.jimenez@depeca.uah.es (D.J.); cobreces@depeca.uah.es (S.C.); losada@depeca.uah.es (C.L.)

**Keywords:** robotics, vision sensor, intelligent spaces

## Abstract

This paper presents a sensor system for robot localization based on the information obtained from a single camera attached in a fixed place external to the robot. Our approach firstly obtains the 3D geometrical model of the robot based on the projection of its natural appearance in the camera while the robot performs an initialization trajectory. This paper proposes a structure-from-motion solution that uses the odometry sensors inside the robot as a metric reference. Secondly, an online localization method based on a sequential Bayesian inference is proposed, which uses the geometrical model of the robot as a link between image measurements and pose estimation. The online approach is resistant to hard occlusions and the experimental setup proposed in this paper shows its effectiveness in real situations. The proposed approach has many applications in both the industrial and service robot fields.

## 1. Introduction

This paper presents a sensor system composed of a single camera attached to a fixed position and orientation in a bounded environment (indoor workplace) which is observing a mobile robot. The aim of the sensor system is to obtain the orientation and position (*i.e.*, pose) of a mobile robot using both visual information retrieved by the camera and relative odometry readings obtained from the internal sensors of the robot. The camera acquisition and image processing tasks are executed in a specialized hardware, which also controls the behavior and internal sensors of the mobile robot through a wireless channel. The proposed schema allows the robot to perform complex tasks without requiring dedicated processing hardware on it. This approach is sustained in the Intelligent Space [1] concept and it can be equally applied to multiple scenarios, specially both in the industrial field (e.g., automatic crane positioning, autonomous car parking) and in the service fields (e.g., wheelchair positioning in medical environments or autonomous driving of mobile platforms). The single camera solution presented in this paper allows to cover large areas with less cameras compared to multiple camera configurations where overlapped areas are mandatory. This feature reduces the cost and improves the reliability of the intelligent space philosophy.

In this paper, we suppose that the camera is correctly calibrated and thus the parameters governing the projection model of the camera are previously known. To connect the pose of the robot with information found in the image plane of the camera, we propose to obtain a 3D geometric model of the mobile robot. Such model is composed of several sparse points whose coordinates represent some well-localized points belonging to the physical structure of the robot. These points are determined by image measurements, called image features, which usually correspond to corner-like points due to texture changes or geometry changes such as 3D vertexes. Usually in industrial fields the image features are obtained by including some kind of artificial marker on the structure of the robot (infrared markers or color bands). These methods are very robust and can be used to recognize human activity and models with high degrees of freedom (AICON 3D online [2] or ViconPeak online systems [3]). However, in this paper we want to minimize the required “a priori” knowledge of the robot, so that it is not necessary to place artificial markers or beacons on it to detect its structure in the images.

Independent of the nature of the image features, the information obtained from a camera is naturally ambiguous and thus some sort of extra metric information are required in order to solve such ambiguity. In this work, we propose to use the odometry sensors inside the robot (*i.e.*, wheel velocities) to act as the metric reference.

**Figure 1.**
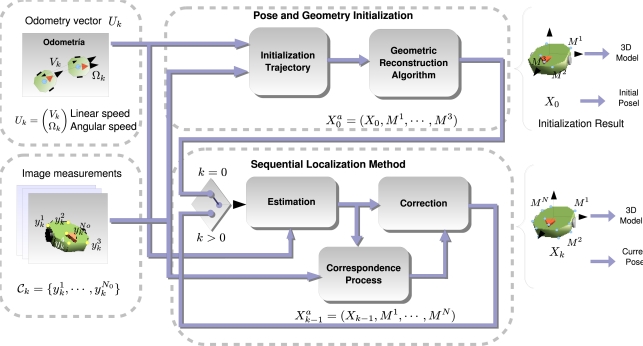
General diagram of the proposed localization system using a vision sensor and odometry readings.

The general diagram of the algorithm proposed in this paper is shown in Figure 1. It shows a clear division of the processes involved in obtaining the pose of the robot: first we denote as “Initialization of Pose and Geometry” to those processes necessary to start up the system, such as the 3D model of the robot and the initial pose it occupies. The initialization consists of a batch processing algorithm where the robot is commanded to follow a certain trajectory so that the camera is able to track some points of the robot’s structure under different viewpoints jointly with the recording of the odometry information. All this information is combined to give the 3D model of the robot and the initial pose it occupies.

Given the initialization information, the second group of processes, named “Sequential Localization”, provides the pose of the robot in a sequential manner. It is composed of a pose estimator, given odometry readings and a pose correction block which combines the estimation of the pose with image measurements to accurately give a coherent pose with the measurements. This algorithm operates entirely on-line and thus the pose is available at each time sample.

Both group of processes are supplied with two main sources of information:

1. Image measurements: they consist of the projection in the camera’s image plane of certain points of the robot’s 3D structure. The measurement process is in charge of searching coherent correspondences through images with different perspective changes due to the movement of the robot.
2. Motion estimation of the robot: The odometry sensors built on-board the robot supply the localization system with an accurate motion estimation in short trajectories but that is prone to accumulative errors in large ones.

### 1.1. Previous Works

Despite the inherent potential of using external cameras to localize robots, there are relative few attempts to solve it compared to the approach that consider the camera on-board the robot [4, 5]. However, some examples of robot localization with cameras can be found in the literature, where the robot is equipped with artificial landmarks, either active [6, 7] or passive ones [8, 9]. In other works a model of the robot, either geometrical or of appearance [10, 11], is learnt previously to the tracking task. In [12, 13], the position of static and dynamic objects is obtained by multiple camera fusion inside an occupancy grid. An appearance model is used afterwards to ascertain which object is each robot. Despite the technique used for tracking, the common point of many of the proposals found in the topic comes from the fact that rich knowledge is obtained previously to the tracking, in a supervised task.

In this paper the localization of the robot is obtained in terms of its natural appearance. We propose a full metric and accurate system based on identifying natural features belonging to the geometric structure of the robot. On most natural objects we can find points whose image projection can be tracked in the image plane independently of the position the object occupies and based on local properties found in the image (*i.e.*, lines, corners or color blobs). Those points are considered natural markers, as they serve as reference points in the image plane that can be easily relate with their three-dimensional counterparts. The set of methods focused on tracking natural markers have become a very successful and deeply studied topic in the literature [14, 15], as they represent the basic measurements of most of existing reconstruction methods.

Scene reconstruction from image measurements is a classic and mature discipline in the computer vision field. Among the wide amount of proposals it can be highlighted those grouped under the name “Bundle Adjustment” [16, 17]. Their aim is essentially to estimate, jointly and optimally, the 3D structure of a scene and the camera parameters from a set of images taken under some kind of motion. (*i.e.*, it can be the camera that moves or equally some part of the scene w.r.t. the camera).

In general terms, Bundle Adjustment reconstruction methods are based in iterative optimization methods which try to minimize the image discrepancy between the measured positions of the 3D model and the expected ones using the last iteration solution. The discrepancy balances the contribution of the measurements into the final solution and plays an important role in this paper. Our main contribution is a redefinition of the discrepancy function using a Maximum Likelihood approach which takes into account the statistical distribution of the error. This distribution is especially affected by the odometry errors which are accumulative in long trajectories.

On the other hand, once a geometric model is obtained using a structure-from-motion approach, its pose with respect to a global coordinate origin can be easily retrieved by measuring the projection of the model in the image plane. This problem, commonly known as the Perspective n Point Problem (PnP), has received considerable attention in the literature, where some accurate solutions are found such as [18] or the recent global solution proposed in [19]. In this paper we instead follow a filtering approach, where not only image measurements but also last time pose information and odometry information are used to obtain the pose. This approach, which is based on the use of a Kalman Filter, is much more regular than solving the PnP problem at each time instant and will be described in this paper.

The paper is organized as follows. In Section 2. the notation and mathematical elements used in the paper are described. In Section 3. the description of the initialization algorithms is given. The online Kalman loop is explained in Section 4. and some results in a real environment are shown in Section 5. Conclusions are in Section 6.

## 2. Definitions and Notation

This section presents a description of the symbols and mathematical models used in the rest of the paper.

### 2.1. Robot and Image Measurements

**Figure 2.**
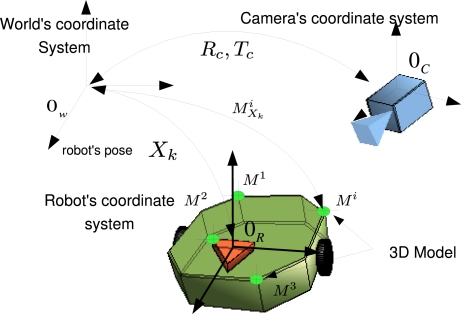
Spatial relationship between world’s coordinate origin *O_W_*, robot’s coordinate origin *O_R_* and camera’s coordinate origin *O_C_*.

The robot’s pose at time *k* is described by a vector *X_k_*. We suppose that the robot’s motion lie on the plane *z* = 0 referred from the world coordinate origin *O_W_* (See Figure 2). The pose vector *X_k_* is described by 3 components (*x_k_, y_k_, α_k_*), corresponding to two position coordinates *x_k_, y_k_* and the rotation angle *α_k_* in the *z* axis. The motion model *X_k_* = *g*(*X*_*k*−1_*, U_k_*) defines the relationship between the current pose *X_k_* with respect to the previous time one *X*_*k*−1_ and the input *U_k_* given by odometry sensors inside the robot. In our proposal the robot used is a differential wheeled platform and thus *U_k_* = (*Vl_k_,* Ω*_k_*)*^T^*, where *Vl_k_* and Ω*_k_* describe the linear and angular speed of the robot’s center of rotation (*O_R_* in Figure 2) respectively. The motion model *g* is then very simple in function of the discretized linear speed *Vl_k_* and the angular speed Ω*_k_*.

The robot’s geometry is composed of a sparse set of *N* 3D points 𝒨 = {*M*^1^, ⋯, *M^N^*} referred from the local coordinate origin *O_R_* described by robot’s pose *X_k_*. The points *M^i^* are static in time due to robot’s rigidness, and thus, no temporal subindex is required for them. Function $M_{X_{k}}^{i}=t\left(X_{k},\;M^{i}\right)$ uses actual pose *X_k_* to express *M^i^* in the global coordinate origin *O_W_* that *X_k_* is referred to (see Figure 2):

(1) $$M_{X_{k}}^{i}=t\left(X_{k},\;M^{i}\right)=R_{k}M^{i}+T_{k}$$

where:

(2) $$R_{k}=\left(\begin{array}{ccc} \text{cos}(\alpha_{k}) & \text{sin}(\alpha_{k}) & 0\\ -\text{sin}(\alpha_{k}) & \cos(\alpha_{k}) & 0\\ 0 & 0 & 1 \end{array}\right)\;\;\;\;\;T_{k}=\left(\begin{array}{c} x_{k}\\ y_{k}\\ 0 \end{array}\right)$$

The augmented vector $X_{k}^{a}$, which is the state vector of the system, is defined as the concatenation in one column vector of both the pose *X_k_* and the set of static structure points 𝒨:

(3) $$X_{k}^{a}={\left({\left(X_{k}\right)}^{T}\;\;\;{\left(M^{1}\right)}^{T}\;\;\;\cdots \;\;\;{\left(M^{N}\right)}^{T}\right)}^{T}$$

An augmented motion model *g^a^* is defined as the transition of the state vector:

(4) $$X_{k}^{a}=g^{a}\left(X_{k-1}^{a},U_{k}\right)={\left({\left(g\left(X_{k-1},U_{k}\right)\right)}^{T}\;\;\;{\left(M^{1}\right)}^{T}\;\;\;\cdots \;\;\;{\left(M^{N}\right)}^{T}\right)}^{T}$$

It must be noticed that the motion model *g^a^* leaves the structure points contained in the state vector untouched, as we suppose that the object is rigid.

The whole set of measurements obtained in the image plane of the camera at time *k* is denoted by *Y_k_*. Each measurement inside *Y_k_* is defined by a two-dimensional vector $y_{k}^{i}={\left(u_{k}^{i}\;\;\;v_{k}^{i}\right)}^{T}$, describing the projection of each point inside 𝒨 in the image plane, where *i* stands for the correspondence with the point *M^i^*. It must be remarked that, in general, *Y_k_* does not contain the image projection of every point in the set *M* as some of them can be occluded depending on the situation.

The camera is modeled with a zero-skew “pin-hole” model (see [17] for more details), with the following matrix *K_c_* containing the intrinsic parameters:

(5) $$K_{c}=\left(\begin{array}{ccc} f_{u} & 0 & u_{0}\\ 0 & f_{v} & v_{0}\\ 0 & 0 & 1 \end{array}\right)$$

The extrinsic parameters of the camera (*i.e.*, position and orientation of the camera w.r.t. *O_W_*) are described using the rigid transformation *R_c_, T_c_* (*i.e.*, rotation matrix and translation vector). The matrix *R_c_* is defined by three Euler angles *α_c_, β_c_, γ_c_*. The vector *T_c_* can be decomposed into its three spatial coordinates *T_x_, T_y_, T_z_*. All calibration parameters can be grouped inside vector *P*:

(6) $$P={\left(f_{u},\;f_{v},\;u_{0},\;v_{0},\;\alpha_{c},\;\beta_{c},\;\gamma_{c},\;T_{x},\;T_{y},\;T_{z}\right)}^{T}$$

Given a single measurement $y_{k}^{i}$ from the set *Y_k_*, its 2D coordinates can be expressed using the “pin-hole” model and the aforementioned calibration parameters:

(7) $$\left(\begin{array}{c} y_{k}^{i}\\ 1 \end{array}\right)=\lambda_{k}^{i}K_{c}\;\left(R_{c}M_{X_{k}}^{i}+T_{c}\right)=\lambda_{k}^{i}K_{c}\;\left(R_{c}R_{k}M^{i}+R_{c}T_{k}+T_{c}\right)$$

where $\lambda_{k}^{i}$ represents a projective scale factor that can be obtained so that the third element of the right part of Equation (7) is equal to one. It is important to remark that the projection model, although simple, depends in a non-linear fashion w.r.t. *M^i^* due to the factor $\lambda_{k}^{i}$. For compactness the projection model of Equation (7) can be described as the function *h*:

(8) $$y_{k}^{i}=h\left(X_{k},\;M^{i},P\right)$$

In the same way, the whole vector *Y_k_* can be expressed with the following function:

(9) $$Y_{k}=h^{a}\left(X_{k}^{a},P\right)={\left(\begin{array}{ccc} h{\left(X_{k},M^{1},P\right)}^{T} & \cdots & h{\left(X_{k},\;M^{N},P\right)}^{T} \end{array}\right)}^{T}$$

### 2.2. Random Processes

This paper explicitly deals with uncertainties by describing every process as a random variable with a Gaussian distribution whose covariance matrix stands for its uncertainty. The random processes are expressed in bold typography (*i.e.*, **X_k_** is the random process describing the pose and *X_k_* is a realization of it) and each of them are defined by a mean vector and a covariance matrix. Therefore $X_{k}^{a}$ is defined by its mean ${\hat{X}}_{k}^{a}$ and covariance $\sum_{k}^{a}$ (or simplifying $𝒩\left({\hat{X}}_{k}^{a},\sum_{k}^{a}\right)$). The following processes are considered in the paper:

- Pose **X_k_** = 𝒩(*X̂_k_*, ∑*_k_*) and 3D model **M** = 𝒩(*M̂*, ∑*_M_*) processes. Its joint distribution is encoded in $X_{k}^{a}=𝒩\left({\hat{X}}_{k}^{a},\sum_{k}^{a}\right)$.
- Measurement process **Y_k_** = 𝒩(*Ŷ_k_,* ∑_*Y*_*k*__), whose uncertainty comes from errors in image detection.
- Odometry input values **U_k_** = 𝒩(*Û_k_*, ∑_*U*_*k*__), which are polluted by random deviations.

## 3. Initialization Process

The initialization step consists of obtaining the initial pose the robot occupies and a 3D model of its structure using visual features (*i.e.*, to obtain an initial guess of $X_{0}^{a}$). The importance of this step is crucial, as the robot’s geometric model serves as the necessary link between robot’s pose and image measurements. The initialization allows to use afterwards the online approach presented in Section 4.

A delayed initialization approach is proposed, based on collecting image measurements and the corresponding odometry position estimation along a sequence of time (*i.e.*, *k* = 1, ⋯, *K*). After taking a sufficient amount of measurements of the object in motion, an iterative optimization method is used to obtain the best solution for $X_{0}^{a}$ according to a cost criterion. The odometry readings are used in this step as metric information, which allows to remove the natural ambiguity produced by measurements from a single camera. The main problem of using odometry as a metric reference for reconstruction comes from the accumulative error it presents, which is a very well-known problem in dead-reckoning tasks.

**Figure 3.**
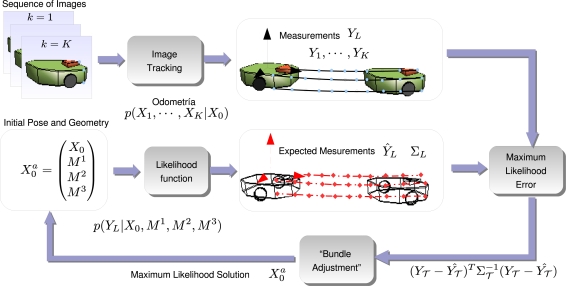
Maximum likelihood initialization by means of reducing the residual of the expected measurements (red diamonds) and the measured image trajectories (blue circles).

The initialization algorithm consists of a Maximum Likelihood (M.L.) estimation of the vector $X_{0}^{a}$, which does not estimate the initialization value itself but its equivalent Gaussian distribution $X_{0}^{a}=𝒩\left({\hat{X}}_{0}^{1},\sum_{0}^{a}\right)$ by using a “Bundle Adjustment” method (See Figure 3). The M.L. approach (see [16, 17] f or more details) allows to properly tackle the growing drift present in the odometry estimation by giving fair less importance or weight to those instants where the odometry is expected to be more uncertain.

In the following sections, the initialization algorithm is explained in detail.

### 3.1. Odometry Estimation

A set of odometry readings, *U*_1_, ⋯, *U_K_* are collected over a set of *K* consecutive time samples *k* = 1, ⋯, *K*, which corresponds to the initialization trajectory performed by the robot. Using a motion model *g* given initial position *X*_0_, an estimation of the robot’s pose in the whole initialization sequence is obtained as follows:

(10) $$\begin{array}{l} X_{1}=g\left(X_{0},U_{1}\right)\\ X_{2}=g\left(X_{1},U_{2}\right)\\ \;\;\;\;\;\;\;\;\;\vdots \\ X_{K}=g\left(X_{K-1},U_{K}\right) \end{array}$$

where we recall that **X_k_** and **U_k_** denote Gaussian processes. Using expression (10), and by propagating the statistical processes through function *g*, the joint distribution *p*(*X*_1_, ⋯, *X_K_*|*X*_0_) can be obtained. The propagation of statistical processes through non-linear functions is in general a very complex task, as it requires to solve integral equations. However in this paper we will follow a first order approximation of the non-linear functions centered in the mean of the process (see [20] for more details). Using this technique the Gaussian processes **X_k_** and **U_k_** can be iteratively propagated through function *g* at the cost of being an approximation. This paper shows that despite being an approximation this approach end in a good estimation of the initialization vector.

By denoting as *X* to the joint vector containing all poses $X={\left(X_{1}^{T},\cdots ,\;X_{K}^{T}\right)}^{T}$, the joint distribution of all poses, given the initial state *X*_0_, *p*(*X*|*X*_0_) is approximated as Gaussian p.d.f. with mean *X̂* and covariance matrix ∑*_X_*.

### 3.2. Image Measurements

**Figure 4.**
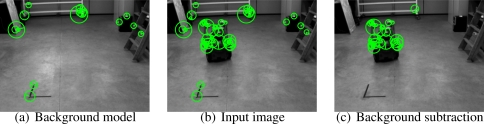
Feature-based background subtraction method.

**Figure 5.**
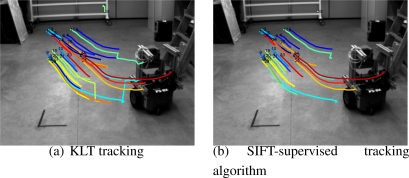
Comparison between the KLT tracker and the SIFT-supervised KLT version.

In this step we describe how to collect the position in the image plane of different points from the robot’s structure during the initialization trajectory. We base our method in the work [15] where the SIFT descriptor is introduced. The process used in the initialization is composed of two steps:

- Feature-based background subtraction.
  We describe the static background of the scene using a single image, from which a set of features, ${𝒡}_{b}=\left\{y_{b}^{1},\cdots ,\;y_{b}^{Nb}\right\}$ and its correspondent SIFT descriptors ${𝒟}_{1}=\left\{d_{1}^{1},\cdots ,\;d_{1}^{Nd}\right\}$ are obtained. The sets 𝒟*_b_* and 𝒡*_b_* are our feature-based background model.
  Given an input image, namely *I_k_*, we find the sets 𝒟*_k_* and 𝒡*_k_*. We consider that a feature $y_{k}^{i}\in {𝒡}_{k}$, $d_{k}^{i}\in {𝒟}_{k}$ belongs to the background if we can find a feature $y_{b}^{i}\in {𝒡}_{b}$, $d_{b}^{j}\in {𝒟}_{b}$ such that $\left|y_{k}^{i}-y_{b}^{j}\right|<R_{\max}$ and $\left|d_{k}^{i}-d_{b}^{j}\right|<d_{\max}$. This method although simple shows to be very effective and robust in real environments (See Figure 4).
- Supervised tracking algorithm.
  Given the first image of the initialization trajectory, namely *I*_1_, we obtain the set of *N* features 𝒡_1_, once they are cleaned by the aforementioned background subtraction method. We propose a method to track them in the whole initialization trajectory.
  By only using SIFT descriptors and its tracking in consecutive frames does not produce stable tracks, specially when dealing with highly irregular objects where many of the features are due to corners. We thus propose to use a classical feature tracking approach based on the Kanade–Lucas–Tomasi (KLT) algorithm [14]. To avoid degeneracy in the tracks, which is a very common problem in those kind of trackers, we use the SIFT descriptors to remove those segments of the tracks that clearly do not correspond to the feature tracked. This can be done easily by checking distance between the descriptors in the track. The threshold used must be chosen experimentally so that it does not eliminate useful parts of the tracks. In Figure 5a we can see the tracks obtained by the KLT tracker without degeneracy supervision. In Figure 5b the automatically removed segments are displayed.

### 3.3. Likelihood Function

Using the feature-based algorithm proposed before a set of measurements in the whole trajectory, *Y*_1_, ⋯, *Y_K_*, is obtained, where each vector *Y_k_* contains the projection of *N* points from robot’s structure at time sample *k*. The set of *N* points, *M*^1^*,* ⋯, *M^N^*, jointly with the initial pose *X*_0_, represent the initialization unknowns. As the robot’s motion does not depend of its structure, the following statistical independence is true:

(11) $$p\left(X_{1},\cdots ,\;X_{K}|X_{0}\right)=p\left(X_{1},\cdots ,\;X_{K}|X_{0},\;M^{1},\cdots ,\;M^{N}\right)=p\left(X_{1},\cdots ,\;X_{K}|X_{0}^{a}\right)$$

If all trajectories are packed into vector *Y_L_*, the following function put in relation *Y_L_* with the distribution of expression (11):

(12) $$Y_{L}=\left(\begin{array}{c} Y_{1}\\ \vdots \\ Y_{K} \end{array}\right)=\left(\begin{array}{c} h^{a}\left(X_{1}^{a},P\right)+V_{1}\\ \vdots \\ h^{a}\left(X_{K}^{a},P\right)+V_{K} \end{array}\right)$$

where **V_k_** represents the uncertainty in image detection. Using distribution showed in (11), and propagating statistics through function (12), the following likelihood distribution is obtained:

(13) $$p\left(Y_{L}|X_{0},\;M^{1},\cdots ,\;M^{N}\right)=p\left(Y_{L}|X_{0}^{a}\right)$$

The likelihood function (13) is represented by a Gaussian distribution using a first order approximation of (12). It is thus defined by a mean *Ŷ_L_* and a covariance matrix ∑*_L_*:

(14) $$p\left(Y_{L}|X_{0}^{a}\right)=\frac{1}{\sqrt{\left|\Sigma_{L}\right|}2^{N}}e^{\frac{1}{2}{\left(Y_{L}-{\hat{Y}}_{L}\right)}^{T}\Sigma_{L}^{-1}\left(Y_{L}-{\hat{Y}}_{L}\right)}$$

### 3.4. Maximum Likelihood Approach

The likelihood function (14), parametrized by its covariance matrix ∑*_L_* and its mean *Ŷ_L_*, is dependent of the conditional parameters and unknown values $X_{0}^{a}$.

The “Maximum Likelihood” estimation consists of finding the values for $X_{0}^{a}$ that maximize the likelihood function:

(15) $$\underset{X_{0},M^{1},\cdots ,M^{N}}{\text{max}}\;p\left(Y_{L}|X_{0},\;M^{1},\cdots ,\;M^{N}\right)$$

In its Gaussian approximation and by taking logarithms, it is converted into the following minimization problem:

(16) $$\underset{X_{0},M^{1},\cdots ,M^{N}}{\text{min}}\;{\left(Y_{L}-{\hat{Y}}_{L}\right)}^{T}\Sigma_{L}^{-1}\left(Y_{L}-{\hat{Y}}_{L}\right)$$

where *Y_L_* is the realization of the process (*i.e.*, the set of measurements from the image) and *Ŷ_L_* are the expected measurements given a value of the parameters *X*_0_*, M*^1^*,* ⋯, *M^N^*.

The configuration of ∑*_L_* is ruled by the expected uncertainty in the measurements and the statistical relation between them. Usually all cross-correlated terms of ∑*_L_* are non-zero, which has an important effect in the sparsity of the Hessian used inside the optimization algorithm and consequently in the computational complexity of the problem (see [21] for more details).

The covariance matrix ∑*_L_* can be approximated assuming either independence between time samples (discarding cross-correlation terms between time indexes) or total independence between time and each measurement (discarding all cross-correlation terms except the 2×2 boxes belonging to a single measurement). In Table 1, the different cost functions are shown depending on the discarded terms of ∑*_L_*. The different approximations of ∑*_L_* have a direct impact in the accuracy and the reconstruction error, and the results will be shown in Section 5.

Intuitively, if ∑*_L_* results to be a identity matrix, the cost function is reduced to a simple image residual minimization extensively used in Bundle Adjustment techniques, where in principle all cost differences $y_{k}^{i}-{\hat{y}}_{k}^{i}$ have equal importance:

(17) $$\underset{X_{0}^{a}}{\text{min}}\sum_{i=1}^{N}\sum_{k=1}^{K_{i}}{\left|\left(y_{k}^{i}-{\hat{y}}_{k}^{i}\right)\right|}^{2}$$

The result of minimizing Equation (16) instead of the usual (17) show significant improvements in the reconstruction error. In Section 5. a comparison is shown between the initialization accuracy under the different assumptions of the matrix ∑*_L_*, from its diagonal version to the complete correlated form. The minimum of (16) is obtained using the Levenberg–Mardquardt [17] iterative optimization method.

We suppose that during the measurement step there is a low probability of encountering outliers in the features. This argument can be very optimistic in some real configurations where more objects appear in the scene and produce occlusions or false matchings. For those cases, all cost functions presented in this section can be modified to be robust against outliers by using M-Estimators. We refer the reader to [17] for more details.

### 3.5. Initialization before Optimization

The use of an iterative optimization method for obtaining the minimum of (16) requires a setup value from which to start iterating. An initial estimation of $X_{0}^{a}$ close to its correct value reduces the probability to fall in a local extrema of the cost function.

The method proposed in this paper to give an initial value for $X_{0}^{a}$ consists of a non-iterative and exact method, which gives directly an accurate solution for $X_{0}^{a}$ in absence of noise in odometry values. This method is based on the assumption that the robot moves in a plane and thus only the angle over its *Z* axis is needed. As explained below, this assumption allows to solve the problem with a rank deficient Linear Least Squares approximation, which is solved using the Singular Value Decomposition of the system’s matrix and imposing a constraint that ensures a valid rotation. Its development is briefly introduced next.

For a point *M^i^* of robot’s model and at time *k* of the initialization trajectory, the image measurement $y_{k}^{i}$ results from the following projection:

(18) $$\left(\begin{array}{c} y_{k}^{i}\\ 1 \end{array}\right)=\lambda_{k}^{i}K_{c}\left(R_{c}\left(R_{k}^{\Delta }R_{0}M^{i}+T_{0}+R_{0}T_{k}^{\Delta }\right)+T_{c}\right)$$

where the matrix $R_{k}^{\Delta }$ and vector $T_{k}^{\Delta }$ represent the rotation and position of the robot in the floor plane given by the odometry supposing that $X_{0}={\left(0\;\;\;0\;\;\;0\right)}^{T}$. The rotation matrix *R*_0_ and the offset *T*_0_ correspond with the initial pose $X_{0}=\left(x_{0}^{1},x_{0}^{2},\alpha_{0}\right)$ in form of rigid transformation:

(19) $$R_{0}=\left(\begin{array}{ccc} \cos\left(\alpha_{0}\right) & -\sin\left(\alpha_{0}\right) & 0\\ \sin\left(\alpha_{0}\right) & \cos\left(\alpha_{0}\right) & 0\\ 0 & 0 & 1 \end{array}\right)\;\;\;\;\;T_{k}=\left(\begin{array}{c} x_{0}^{1}\\ x_{0}^{2}\\ 0 \end{array}\right)$$

where *R*_0_ is a non-linear function of the orientation angle. The expression (18) depends non-linearly of vector $X_{0}^{a}$ and so a new parametrization is proposed jointly with a transformation which removes the projective parameter $\lambda_{k}^{i}$.

The point *M^i^* is replaced by the rotated $M_{X_{0}}^{i}=R_{0}M^{i}$, removing thus the product between unknowns. The orientation angle in the pose is substituted by parameters *a* = *cos*(*α*_0_) and *b* = *sin*(*α*_0_), with the constraint *a*^2^ + *b*^2^ = 1. Using the new parameterization the expression (18) is transformed in the following:

(20) $$\left(\begin{array}{c} y_{k}^{i}\\ 1 \end{array}\right)=\lambda K_{c}\left(R_{c}\left(R_{k}^{\Delta }M_{X_{0}}^{i}+T_{0}+L_{T_{k}^{\Delta }}\left(\begin{array}{c} a\\ b\\ 0 \end{array}\right)\right)+T_{c}\right)$$

with $L_{T_{k}^{\Delta }}$:

(21) $$L_{T_{k}^{\Delta }}=\left(\begin{array}{ccc} x_{k}^{1,\Delta } & -x_{k}^{2,\Delta } & 0\\ -x_{k}^{2,\Delta } & x_{k}^{1,\Delta } & 0\\ 0 & 0 & 1 \end{array}\right)$$

where $x_{k}^{1,\Delta }$ y $x_{k}^{2,\Delta }$ are the two first coordinates of $T_{k}^{\Delta }$.

The new unknown vector, which correspond to the new parametrization of $X_{0}^{a}$, is:

(22) $$\Phi ={\left(\begin{array}{ccccc} x_{0}^{1} & x_{0}^{2} & a & b & {\left(M_{X_{0}}^{1}\right)}^{T},\cdots ,{\left(M_{X_{0}}^{N}\right)}^{T} \end{array}\right)}^{T}$$

The expression (20) is decomposed in the following elements:

(23) $$\left(\begin{array}{c} u_{k}^{i}\\ v_{k}^{i} \end{array}\right)=y_{k}^{i}\;\;\;\;\;\left(\begin{array}{c} U_{k}^{i}\\ V_{k}^{i}\\ S_{k}^{i} \end{array}\right)=K\left(R_{c}(R_{k}^{\Delta }M_{X_{0}}^{i}+T_{0}+L_{T_{k}^{\Delta }})+T_{c}\right)$$

where $U_{k}^{i}$, $V_{k}^{i}$ y $S_{k}^{i}$ are linear in terms of Φ but not in terms of $y_{k}^{i}$.

(24) $$U_{k}^{i}=L_{U_{k}}^{i}\Phi +b_{U_{k}}^{i}\;\;\;\;\;\;V_{k}^{i}=L_{V_{k}}^{i}\Phi +b_{V_{k}}^{i}\;\;\;\;\;\;S_{k}^{i}=L_{S_{k}}^{i}\Phi +b_{S_{k}}^{i}$$

The projective scale is removed from the transformation by using vector product properties:

(25) $$\left(\begin{array}{c} u_{k}^{i}\\ v_{k}^{i}\\ 1 \end{array}\right)=\lambda_{k}^{i}\left(\begin{array}{c} U_{k}^{i}\\ V_{k}^{i}\\ S_{k}^{i} \end{array}\right)\to \left(\begin{array}{c} u_{k}^{i}\\ v_{k}^{i}\\ 1 \end{array}\right)\times \left(\begin{array}{c} U_{k}^{i}\\ V_{k}^{i}\\ S_{k}^{i} \end{array}\right)=\left(\begin{array}{c} 0\\ 0\\ 0 \end{array}\right)$$

where two lineally independent equations are obtained for Φ.

(26) $$\{\begin{array}{l} v_{k}^{i}S_{k}^{i}-V_{k}^{i}=0\\ -u_{k}^{i}S_{k}^{i}+U_{k}^{i}=0 \end{array}\;\;\;\;\;\;\to \{\begin{array}{l} \left(v_{k}^{i}L_{S_{k}}^{i}-L_{V_{k}}^{i}\right)\Phi =v_{k}^{i}b_{S_{k}}^{i}-b_{V_{k}}^{i}\\ \left(-u_{k}^{i}L_{S_{k}}^{i}+L_{U_{k}}^{i}\right)\Phi =-u_{k}^{i}b_{S_{k}}^{i}+b_{U_{k}}^{i} \end{array}$$

Using all measurements inside *Y_L_*, a lineal system of equations is obtained in terms of Φ:

(27) $$A\Phi =B\;\;\;\;\;\;A=\left(\begin{array}{c} \left(v_{1}^{1}L_{S_{1}}^{1}-L_{V_{1}}^{1}\right)\\ \left(-u_{k}^{1}L_{S_{1}}^{1}+L_{U_{1}}^{1}\right)\\ \vdots \\ \left(v_{K}^{N}L_{S_{K}}^{N}-L_{V_{K}}^{N}\right)\\ \left(-u_{K}^{N}L_{S_{K}}^{N}+L_{U_{K}}^{N}\right) \end{array}\right)\;\;\;\;\;\;B=\left(\begin{array}{c} v_{1}^{1}b_{S_{1}}^{1}-b_{V_{1}}^{1}\\ -u_{1}^{1}b_{S_{1}}^{1}+b_{U_{1}}^{1}\\ \vdots \\ v_{K}^{N}b_{S_{K}}^{N}-b_{V_{K}}^{N}\\ -u_{K}^{N}b_{S_{K}}^{N}+b_{U_{K}}^{N} \end{array}\right)$$

It is straightforward to show that system of (27) has a single-parameter family of solutions. If Φ_0_ is a possible solution, then Φ_0_ + *ψ*Δ is a solution for any *ψ* ∈ *R*, with Δ:

(28) $$\Delta =\left(\begin{array}{ccccccc} T_{c,x^{1}} & T_{c,x^{2}} & a & b & {\left(\begin{array}{c} 0\\ 0\\ T_{c,x^{3}} \end{array}\right)}^{T} & \cdots & {\left(\begin{array}{c} 0\\ 0\\ T_{c,x^{3}} \end{array}\right)}^{T} \end{array}\right)$$

In fact, if Δ is normalized, it matches up with the eigen-vector associated to the zero eigenvalue of matrix *A^T^ A*.

Using the constraint that *a*^2^ + *b*^2^ = 1, and the singular value decomposition of matrix *A*, an exact solution of system (20) is obtained.

Once Φ is available, the solution $X_{0}^{a}$ is obtained by inverting the parametrization:

(29) $$\begin{array}{ccc} \alpha_{0}={\text{tan}}^{-1}(a,b) & M^{i}=R_{0}^{-1}M_{0}^{i} & X_{0}^{a}={\left(\begin{array}{cccccc} x_{0}^{1} & x_{0}^{2} & \alpha_{0} & {\left(M^{1}\right)}^{T} & \cdots & {\left(M^{N}\right)}^{T} \end{array}\right)}^{T} \end{array}$$

This method, although exact, is prone to error due to odometry noise and does not benefit from the Maximum Likelihood metric. However it is valid as a method to give an initial value for $X_{0}^{a}$ before using the iterative approach.

### 3.6. Degenerate Configurations

The kind of trajectory performed by the robot during initialization has direct influence in the solution of $X_{0}^{a}$. There are three kinds of movements that yields degenerate solutions:

- Straight motion: there is no information about the center of rotation of the robot and thus the pose has multiple solutions.
- Rotational motion around robot axis: the following one-parameter (*i.e.*, *n*) family of solutions gives identical measurements in the image plane:
  (30) $$M_{n}^{i}={nM}^{i}+(n-1)\left(\begin{array}{c} 0\\ 0\\ {\bar{T}}_{c}(3) \end{array}\right)\;\;\;\;\;T_{0_{n}}={nT}_{0}+(n-1)\left(\begin{array}{c} {\bar{T}}_{c}(1)\\ {\bar{T}}_{c}(2)\\ 0 \end{array}\right)$$
  with ${\bar{T}}_{c}=R_{c}^{T}T_{c}$.
  (31) $$X_{k}^{a}(n)=\left(\begin{array}{ccccc} T_{0_{n}} & \alpha_{0} & M_{n}^{1} & \cdots & M_{n}^{N} \end{array}\right)$$
- Circular trajectory: under a purely circular trajectory the following one-parameter family of initialization vectors gives identical measurements in the image plane:
  (32) $$M_{n}^{i}={nM}^{i}+R_{0}^{T}\;\;\;T_{0_{n}}=nT_{0}+(n-1)R_{c}^{T}T_{c}$$
  (33) $$X_{k}^{a}(n)=\left(\begin{array}{ccccc} T_{0_{n}} & \alpha_{0} & M_{n}^{1} & \cdots & M_{n}N \end{array}\right)$$

In practical cases it has been proved to be effective enough to combine straight trajectories with circular motion to avoid degeneracies in the solution of $X_{0}^{a}$.

### 3.7. Obtaining the Gaussian Equivalent of the Solution

Once the minimum of (16) is reached we suppose that the resulting value of $X_{0}^{a}$ is the mean of the distribution $X_{0}^{a}$. This section describes how to also obtain the covariance matrix.

The covariance matrix $\sum_{0}^{a}$ of the optimized parameters is easily obtained by using a local approximation of the term *Y_L_* − *Ŷ_L_* in the vicinity of the minimum ${\hat{X}}_{0}^{a}$ using the following expression:

(34) $$\sum_{0}^{a}={\left(J^{T}\sum_{L}^{-1}J\right)}^{-1}$$

where *J* is the Jacobian matrix of *Ŷ* with respect to parameters $X_{0}^{a}$. The Jacobian is available from the optimization method, in which is used to compute the iteration steps.

## 4. Online Robot Localization

In this section the solution to $X_{k}^{a}$ given the last pose information is derived. The fact that last frame information is available and the assumption of soft motion between frames allows to greatly simplify the problem.

**Figure 6.**
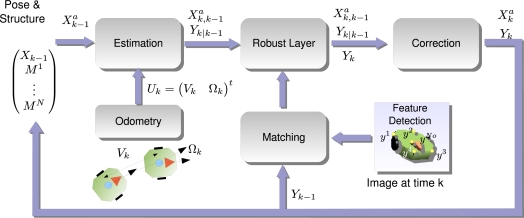
Overview of the online algorithm.

A special emphasis is given to the fact that any process handled by the system is considered a random entity, in fact a Gaussian distribution defined at each case by its mean vector and covariance matrix. The problem of obtaining pose and structure, encoded in $X_{k}^{a}$ given image observations *Y_k_* and the previous time estimation $X_{k-1}^{a}$ is viewed from the point of view of statistical inference, which means searching for the posterior probability distribution $p\left(X_{k}^{a}|Y_{1},\cdots ,Y_{k}\right)$. That distribution gives the best estimation of $X_{k}^{a}$ given all the past knowledge available. In Figure 6, a brief overview of the online method is presented.

The online approach is divided into three steps:

- **Estimation Step**: using the previous pose distribution $p\left(X_{k-1}^{a}|Y_{1},\cdots ,Y_{k-1}\right)$, defined by its mean ${\hat{X}}_{k-1}^{a}$ and covariance matrix $\sum_{k-1}^{a}$, and the motion model *g^a^*, a Gaussian distribution which infers the next state is given $p\left(X_{k}^{a}|Y_{1},\cdots ,Y_{k-1}\right)$.
- **Robust Layer**: the correspondence between image measurements and the 3D model of the robot easily fails, so a number of wrongly correspondences or outliers pollute the measurement vector *Y_k_*. Using a robust algorithm and the information contained in the state vector, the outliers are discarded before the next step.
- **Correction Step**: using an outlier-free measurement vector, we are confident to use all the information available to obtain the target posterior distribution $p\left(X_{k}^{a}|Y_{1},\cdots ,Y_{k}\right)$

In all three steps it is required to propagate statistic processes over non-linear functions (*f* and *h*). As was stated in the previous section we show how to face the problem using first order expansions as it offers more compactness and is more readable. As a consequence the “Estimation” and “Correction” steps are solved using the so called Extended Kalman Filter (EKF) equations, which have been already implemented in problems of similar complexity such as visual Simultaneous Localization and Mapping (SLAM) [5].

### 4.1. Estimation Step

The estimation step uses the motion models available to infer the next pose of the robot which implies an increment in uncertainty. Starting from the last pose distribution $p\left(X_{k-1}^{a}|Y_{1},\cdots ,Y_{k-1}\right)=N\left({\hat{X}}_{k-1}^{a},\sum_{k-1}^{a}\right)$, the motion model *g^a^* and the noise included in odometry values, the following update is obtained:

(35) $$\begin{array}{l} {\hat{X}}_{k|k-1}^{a}=g^{a}\left({\hat{X}}_{k}^{a},U_{k}\right)\\ \sum_{k|k-1}^{a}=J_{x}^{T}\sum_{k-1}^{a}J_{x}+J_{U}^{T}\sum_{W}J_{U}, \end{array}$$

where ${\hat{X}}_{k|k-1}^{a}$ and $\sum_{k|k-1}^{a}$ are the mean and covariance matrix of distribution:

$$p\left(X_{k}^{a}|Y_{1},\cdots ,Y_{k-1}\right)$$

The matrices *J_X_* and *J_U_* are the first derivatives of the function *g^a^* with respect to $X_{k-1}^{a}$ and *U_k_* respectively. Usually *J_X_* in odometry systems is the identity, therefore, at this step, the covariance matrix $\sum_{k|k-1}^{a}$ results to be bigger in terms of eigenvalues, which means uncertainty.

### 4.2. Correction Step

The correction step removes the added uncertainty in the estimation by using image measurements. It passes from the distribution $p\left(X_{k}^{a}|Y_{1},\cdots ,Y_{k-1}\right)$ to the target distribution $p\left(X_{k}^{a}|Y_{1},\cdots ,Y_{k}\right)$, which includes the last measurement.

Using the estimation shown in (35), and knowing the correspondence between measurements with the camera and structure point of the state vector, the estimated measurement is given:

(36) $$Y_{k|k-1}=h^{a}\left(X_{k}^{a}\right)$$

(37) $$\sum_{Y_{k|k-1}}=J_{h}^{T}\sum_{k|k-1}^{a}J_{h}+\sum_{V}$$

(38) $$\sum_{X^{a}Y}=\sum_{k|k-1}^{a}J_{h}$$

where *J_h_* is the Jacobian matrix of the function *h^a^* with respect to $X_{k}^{a}$ and ∑*_V_* is block diagonal matrix with ∑*_v_* on each block. Function *h^a^* performs the projection in the image plane of the camera of all visible points that form up the measurement vector *Y_k_*. The correction step itself is a linear correction of $X_{k|k-1}^{a}$ and $\sum_{k|k-1}^{a}$ by means of the Kalman gain *K_G_*:

(39) $$K_{G}=\sum_{X^{a}Y}\sum_{Y_{k|k-1}}^{-1}$$

(40) $$X_{k}^{a}=X_{k|k-1}^{a}+K_{G}\left(Y_{k}-Y_{k|k-1}\right)$$

(41) $$\sum_{k}^{a}=\sum_{k|k-1}^{a}-K_{G}\sum_{X^{a}Y}^{T}$$

As it is stated in (41) the resulting $\sum_{k}^{a}$ is reduced compared to $\sum_{k|k-1}^{a}$ which means that after the correction step, the uncertainty is “smaller”.

### 4.3. Robust Layer

The robust layer has the objective of removing bad measurements from *Y_k_* to avoid inconsistent updates of $X_{k}^{a}$ in the correction step. In this paper we propose to include the Random Sample Consensus (RANSAC) algorithm [22] between the estimation and correction step of the filter. The general idea is to found among the measured data *Y_k_* a set which agrees in the pose *X_k_*, using the 3D model obtained in $X_{k-1}^{a}$.

The interest of applying RANSAC in a sequential update approach resides on several reasons: firstly it allows to efficiently discard outliers from *Y_k_* preventing algorithm’s degeneracy, which happens even if the motion model is accurate. Secondly, compared to online robust approaches, where a robust cost function is optimized, RANSAC allows not to break the Kalman filter approach, as it only cleans the measurement vector of outliers. Furthermore we have observed experimentally that the RANSAC algorithm can be very fast between iterations, as only a few outliers are inside the data. (We use the RANSAC implementation described in [17], which implements a dynamical computation of the outlier probability).

The RANSAC method proposed in the commented framework obtains the consensus pose *X_k_* from the set of measurements *Y_k_* and the 3D model available in $X_{k-1}^{a}$ using the algorithm presented in [18]. For a robot moving in a plane, as it is the case with the mobile robot considered in this paper, it is enough to use a minimum of 2 correspondences between the model and the measurement which makes the RANSAC very fast for removing outliers.

### 4.4. Image Measurements

Contrary to the initialization case, in this step we have an accurate prediction of the tracked points in the image plane at each time instant, namely vector *Y*_*k*|*k*−1_. Using such prediction we can easily match the 3D points in the state vector with measurements taken in a measurement set 𝒡*_k_*, using the SIFT detector applied to current image *I_k_*. The matching is done in terms of distance in the image plane. Let $y_{k|k-1}^{i}$ a feature inside vector *Y*_*k*|*k*−1_ and $y_{k}^{j}$ a candidate obtained using SIFT method. We conclude that they are matched if ${\left|y_{k}^{j}-y_{k|k-1}^{i}\right|}_{M}^{2}<\tau_{\max}$, where ‖*_M_* states for the Mahalanobis distance using the covariance ∑_*y*_*k*|*k*−1__ computed from the matrix ∑_*Y*_*k*|*k*−1__. The Mahalanobis distance allows to take into account the uncertainty predicted for $y_{k|k-1}^{i}$ and also helps to select a threshold *τ_max_* with a probabilistic criterion.

## 5. Results

This section describes the experimental setup developed to support the theoretical algorithms proposed in this paper. The experiments are divided in those performed over synthetic data and those run in a real implementation of the Intelligent Space in the University of Alcala (ISPACE-UAH) [23]. In both kind of experiments the same camera parameters are used, derived from the real device used in its real placement. The single camera consists of a CCD based sensor with resolution of 640 × 480 pixels and a physical size of 1/2 (around 8 mm diagonal). The optical system is chosen with a focal length of 6.5 mm which gives around 45° of Field of View (FOV). The camera is connected to a processing and acquisition node through a IEEE1394 port, which support 15 fps in RGB image format acquisition. The intrinsic parameters are the following:

(42) $$\begin{array}{cccc} f_{u}=636.7888 & f_{v}=637.5610 & u_{0}=313.3236 & v_{0}=210.6894 \end{array}$$

**Figure 7.**
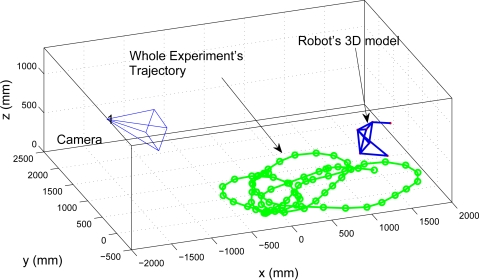
Geometric distribution of the camera and robot’s trajectory.

The camera is placed with respect a global coordinate origin, as it is displayed in Figure 7. Camera calibration is performed previously to the localization task, using checkerboards as calibration patterns and the “Matlab Calibration Toolbox” implemented by [24]. The distortion parameters of the camera are not considered in this case. As it can be shown in Figure 7, the camera is placed in oblique angle, which is specially useful for covering large areas with less FOV requirements (less distortion) compared to overhead configurations.

The robotic platform is connected to the same processing node controlling the camera by means of a wireless network. The camera acquisition and the odometry values obtained from the robot are synchronized. The control loop of the robot is internal, and it is prepared to follow position landmarks based on odometry feedback at faster sampling frequency than the image localization system (15 fps). Therefore, for each image acquisition cycle, the odometry system obtain several readings that are used by the internal loop control. In this paper the localization information provided by the cameras is not included in the control loop of the robot.

### 5.1. Simulated Data

**Figure 8.**
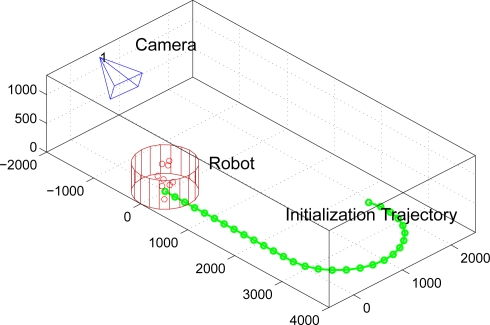
Initialization trajectory of the robot in the experiment based on synthetic data.

In this experiment the robot structure is simulated with a set of *N* = 10 points distributed randomly inside a cylinder with radius *R* = 0.5*m* and height *h* = 1*m*. The odometry system is supposed to have an uncertainty $\sigma_{\mathrm{Vl}}^{2}=10$ and $\sigma_{\Omega }^{2}=1$ in linear and angular speed respectively. The initialization trajectory is shown in Figure 8. The image measurements are considered polluted with a Gaussian process of $\sigma_{v}^{2}=10$, independently of each measurement and image coordinate.

**Figure 9.**
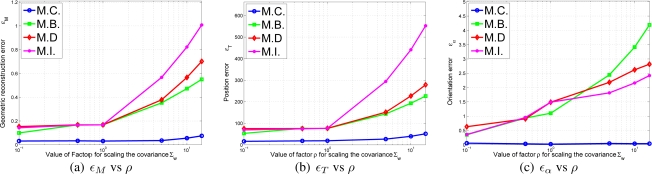
Experiment showing the different initialization errors in function of the amount of error in odometry readings.

**Figure 10.**
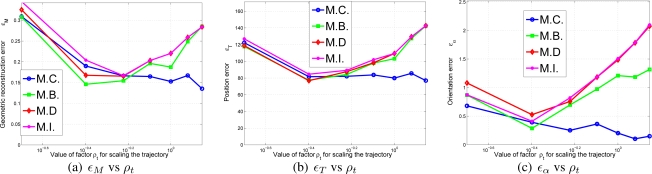
Experiment showing the different initialization errors in function of the trajectory length performed by the robot.

To compare the performance of the initialization method proposed in Section 3., the following error magnitudes are described:

(43) $${ɛ}_{M}=\frac{\sqrt{\sum_{i=1}^{N}{\left\|M^{i}-M_{\mathrm{gth}}^{i}\right\|}^{2}}}{\sqrt{\sum_{i=1}^{N}{\left\|M_{\mathrm{gth}}^{i}\right\|}^{2}}}\;\;\;{ɛ}_{T}=\left\|T_{0}-T_{0,\mathrm{gth}}\right\|\;\;\;{ɛ}_{\alpha }=\;\left\|\alpha_{0}-\alpha_{0,\mathrm{gth}}\right\|$$

where *T*_0*,gth*_*, α*_0*,gth*_ and $M_{\mathrm{gth}}^{i}$ correspond to the ground truth values of the initialization vector $X_{0}^{a}$. The following two experiments are proposed:

- *Initialization errors in function of the odometry error*. In this experiment the value of $\sigma_{\mathrm{Vl}}^{2}$ and $\sigma_{\Omega }^{2}$ are multiplied by the following multiplicative factor:
  (44) $$\rho \in \left(\begin{array}{cccccc} 0.01 & 0.1 & 0.5 & 1 & 5 & 10 \end{array}\right)$$
  The different errors of Equation 43 can be viewed in Figure 9a–c in terms of *ρ*. As it can be observed in the results, the M.C. (Complete correlated matrix ∑*_L_*) method outperforms the rest of approximations of ∑*_L_*, specially the full diagonal method M.I., which means that the statistical modelling proposed in this paper is effective.
- *Initialization errors in function of the trajectory length*. The trajectory used in the experiment and displayed in Figure 8 is uniformly scaled by parameter *ρ_t_*,
  (45) $$\rho_{t}\in \left(\begin{array}{ccccccc} 0.2 & 0.4 & 0.6 & 0.8 & 1 & 1.2 & 1.4 \end{array}\right)$$
  so that it can be guessed the relationship between trajectory length and initialization errors. In Figure 10 the initialization errors are displayed versus *ρ_t_*.
  In light of the results shown in Figure 10a–c, the M.C. method is capable of reducing the error no matter how large is the trajectory chosen. However, in the rest of the approximations of ∑*_L_* there is an optimal point where the initialization errors are minimum. This results make sense, as without statistical modelling large trajectories contain accumulative errors which usually affects the final solution of $X_{0}^{a}$.

Both experiments clearly manifest that the complete matrix ∑*_L_*, with all its cross-correlated terms (M.C.), outperforms the rest of proposals, especially when it is compared to the case where ∑*_L_* is the identity matrix (M.I.), which means no statistical modeling.

### 5.2. Real Data

**Figure 11.**
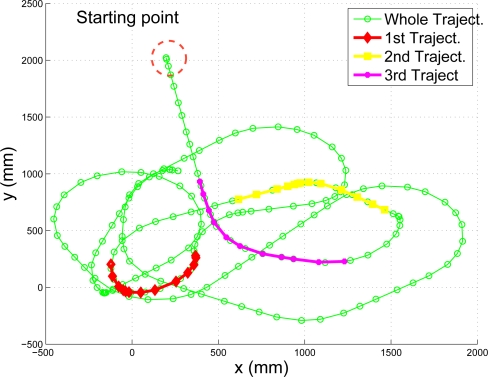
Intervals of robot’s trajectory used for initialization.

The initialization experiment proposed in this paper consists of a robot performing a short trajectory from which its 3D model and initial position is obtained using the results of Section 3.. We present a comparison of the initialization results using three different trajectories. Each one of the trajectories is displayed in Figure 11 as a colored interval of the whole trajectory performed by the robot in the experiment.

**Figure 12.**
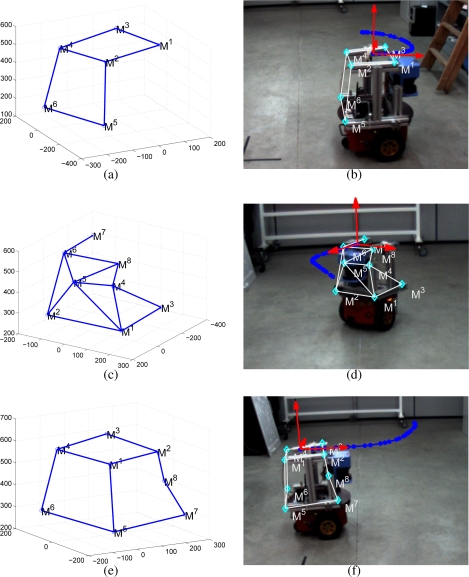
Single camera initialization results. On each row it is shown the resulting reconstruction and its projection in the image plane of the camera.

The results of the initialization method on each of the 3 trajectories selected is shown in Figure 12. Depending on the trajectory used, the features viewed by the camera vary and thus the corresponding initialized 3D model. As it can be seen in Figure 12, the 3D model is accurate and its projection matches with points in the robot’s structure in all cases. It must be remarked that on each case, the position obtained as a result of the initialization (*i.e.*, *X*_0_) corresponds to the first position of each interval.

**Figure 13.**
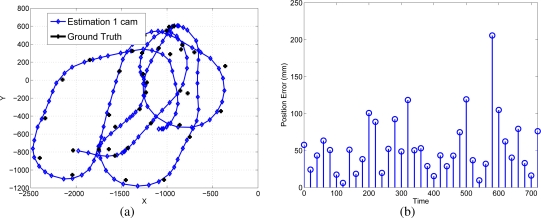
Online robot’s pose compared with the “ground-truth”.

The sequential algorithm is tested using the whole trajectory shown in Figure 11. The initial pose and 3D model are the result of the initialization results shown in Figure 12e,f. The estimated position of the robot, compared to a “ground truth” measurement, is presented in Figure 13a,b.

The ground truth is obtained by means of a manually measured 3D model of the robot. The model is composed of 3D points, that are easily recognized by a human observer. By manually clicking points of the 3D model in the image plane, and by using the method proposed in [18], the reference pose of the robot is obtained in some of the frames of the experiment.

**Figure 14.**
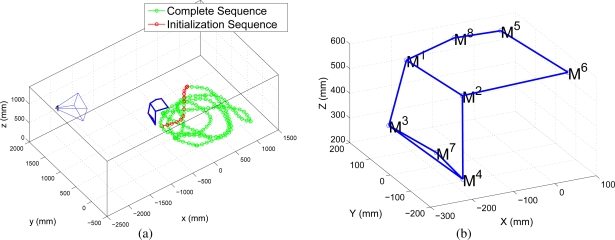
Scene geometry and 3D model obtained during initialization.

Another experiment is proposed to test the online algorithm with occlusions and a larger path followed by the robot. In Figure 14a it is shown the geometric placement of the camera and in Figure 14b it is shown the geometric model obtained during initialization.

**Figure 15.**
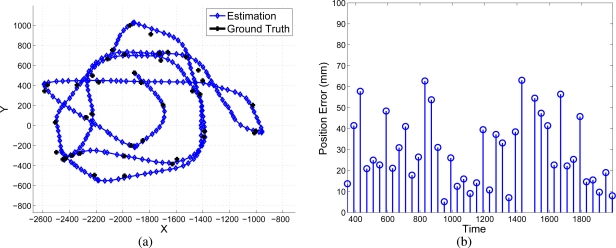
Localization results in an experiment with occlusions.

**Figure 16.**
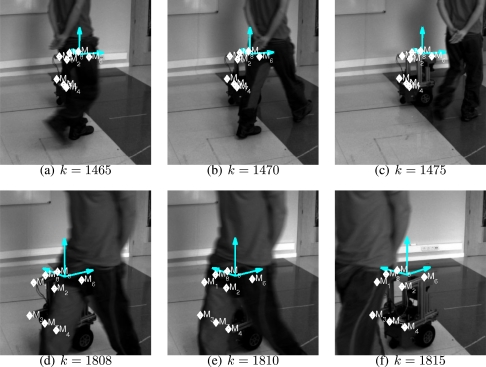
Occlusions and its influence in the localization accuracy.

The resulting localization error is shown in Figure 15b. In Figure 16 it is shown several frames, indexed by the time sample number *k*, presenting hard occlusions between the camera and the robot without losing the tracking. The RANSAC method used in the Kalman loop avoid erroneous matches in the occluded parts of the object. Besides, in those frames with completely occluded features, only the estimation step of the Kalman filter is done, which is accurate enough for short periods of time.

In both sequential experiments the 3D model is composed of 8 to 10 points and no extra geometrical information is introduced in the state vector. As a future line of study a simultaneous reconstruction and localization approach can be adopted to introduce online extra 3D points in the state vector as the robot is tracked. A very similar approach is done in the Simultaneous Localization and Mapping (SLAM) problem, and some of their solutions [5] are perfectly applicable to the problem assessed in this paper.

## 6. Conclusions

This paper has presented a complete localization system for mobile robots inside intelligent environments with a single external camera. The objectives of obtaining the pose of the robot based on its natural appearance has been tackled in the paper using a reconstruction approach followed by a sequential localization approach.

The main contributions of this paper are summarized in the following list:

- The initialization step provides a non-supervised method for obtaining the initial pose and structure of the robot previously to its sequential localization. A Maximum Likelihood cost function is proposed, which obtains the pose and geometry given a trajectory performed by the robot. The proposal of this paper allows to compensate for the odometry drift in the solution. Also an exact initialization method has been proposed and the degenerate configurations have been identified theoretically.
- The online approach of the algorithm obtains the robot’s pose by using a sequential Bayesian inference approach. A robust step, based on the RANSAC algorithm, is proposed to clean the measurement vector out of outliers.
- The results show that the proposed method is suitable to be used in real environments. The accuracy and non-drifting nature of the pose estimation have been also evaluated in a real environment.

The future research must be oriented to scale the problem for large configurations of non-overlapped cameras and multiple robots, where extra problems arise, such as to automatically detect the transitions between cameras and online refinement of the geometric models. It is important from our point of view to, in the future, combine the information given by the system proposed in this paper with information sensed onboard the robots using cameras. This approach allows to jointly build large maps attached to information given by the cameras, so that robots can be localized and controlled to perform a complex task.

## Figures and Tables

**Table 1.** Different cost functions depending on the approximations of ∑*_L_*.

Type of Approximation	cost function
Complete Correlated matrix ∑*_L_* (M.C.)	${\left(Y_{L}-{\hat{Y}}_{L}\right)}^{T}\sum_{L}^{-1}\left(Y_{L}-{\hat{Y}}_{L}\right)$
2*N* × 2*N* block approximation of ∑*_L_* (M.B.)	$\sum_{k=1}^{K}{\left(Y_{k}-{\hat{Y}}_{k}\right)}^{T}\sum_{Y_{k}}^{-1}\left(Y_{k}-{\hat{Y}}_{k}\right)$
2 × 2 block approximation of ∑*_L_* (M.D.)	$\sum_{k=1}^{K}\sum_{i=1}^{N}{\left(y_{k}^{i}-{\hat{y}}_{k}^{i}\right)}^{T}\sum_{y_{k}^{i}}^{-1}\left(y_{k}^{i}-{\hat{y}}_{k}^{i}\right)$
Identity approximation of ∑*_L_* (M.I.)	$\sum_{k=1}^{K}\sum_{i=1}^{N}{\left(y_{k}^{i}-{\hat{y}}_{k}^{i}\right)}^{T}\left(y_{k}^{i}-{\hat{y}}_{k}^{i}\right)$
